# Cultured microbial community complexity is associated with antimicrobial resistance and Enterobacterales enrichment in adult odontogenic infections

**DOI:** 10.1007/s10006-026-01563-3

**Published:** 2026-04-24

**Authors:** Alina Marie Schmitz, N. L. Kern, H. Ward, M. Sauerbrey, F. Mrosk, O. Wagendorf, S. Nahles, C. Rendenbach, N. Neckel, M. Heiland, S. Koerdt

**Affiliations:** Department of Oral and Maxillofacial Surgery, Charité – Universitätsmedizin Berlin, corporate member of Freie Universität Berlin and Humboldt-Universität zu Berlin, Augustenburger Platz 1, Berlin, 13353 Germany

**Keywords:** Odontogenic infection, Antimicrobial resistance, Immunosuppresison, Polymicrobial infection, Microbial complexity

## Abstract

**Background:**

Odontogenic infections are polymicrobial entities with increasing antimicrobial resistance. Although immunosuppression is commonly considered a major risk factor for resistant and complex infections, concepts from microbial ecology suggest that community complexity itself may be associated with distinct microbial phenotype and susceptibility patterns.

**Methods:**

We performed a retrospective cohort study of consecutive patients undergoing surgical treatment for odontogenic infections at a tertiary maxillofacial center between 2017 and 2022. Intraoperative specimens were processed using standardized aerobic and anaerobic culture protocols. Primary outcomes were polymicrobial infection, *Enterobacterales* detection, and antimicrobial resistance; *MRSA* and *Candida spp*. were secondary outcomes. Immunosuppression was defined by systemic immunosuppressive therapy, chemotherapy, solid organ transplantation, or HIV infection. Cultured microbial community complexity was operationalized as the number of distinct pathogens detected by routine culture in each patient.

**Results:**

Among 695 included patients, polymicrobial infection was detected in 62.0% and antimicrobial resistance in 40.6%, without differences by immune status. Enterobacterales were more frequently detected in immunosuppressed patients (13.9% vs. 3.9%). Unsupervised clustering of pathogen profiles did not segregate by immune status. High microbial complexity was associated with a distinct microbiological profile associated with near-universal polymicrobial infection (97.2% vs. 55.3%), increased antimicrobial resistance (72.9% vs. 34.8%), and marked *Enterobacterales* enrichment (19.6% vs. 2.9%) (all *p* < 0.001). In fully adjusted models, higher cultural microbial complexity showed the strongest adjusted association with all adverse microbiological outcomes, whereas no independent association was observed for the broad immunosuppression variable used in this study. Dose–response analyses demonstrated a graded increase in *Enterobacterales* detection and antimicrobial resistance with rising pathogen burden.

**Conclusions:**

In this retrospective cohort of surgically treated odontogenic infections, higher cultured microbial complexity was strongly associated with polymicrobial infection, Enterobacterales detection, and antimicrobial resistance. These findings support support further investigation of community-level microbiological features for future risk stratification approaches.

**Supplementary Information:**

The online version contains supplementary material available at 10.1007/s10006-026-01563-3.

## Introduction

Odontogenic infections are common maxillofacial emergencies and are typically characterized by polymicrobial communities with frequent anaerobic involvement and increasing antimicrobial resistance. Clinical management is substantially complicated by complex microbial profiles, gram-negative shifts and resistant pathogens 1, 2, 3, 4, 5, 6. Several host-related factors, such as age-related oral microbiota dysbiosis, altered mucosal immunity, prior antibiotic exposure, and hospitalization, have been associated with shifts toward complex microbial profiles, gram-negative species, and resistant pathogens 7 ,8, 9, 10, 11, 12.

Beyond host-related factors, community structure and ecological interactions may also influence infection behavior and antimicrobial susceptibility 8 .

Immunosuppressed patients are traditionally regarded as particularly vulnerable. Impaired mucosal defense and reduced microbial control are believed to facilitate dysbiosis, polymicrobial overgrowth, and potentially resistant infections, while blunted clinical symptoms may delay diagnosis and increase the risk of deep neck involvement or systemic complications 9, 10, 11, 12, 13, 14, 15, 16, 17. Early surgical drainage and microbiological sampling are therefore emphasized, yet it remains uncertain whether immune status truly determines the microbial phenotype in odontogenic infections 18, 19 .

Emerging concepts from microbial ecology suggest that community-level microbial organization may be relevant to infection behavior and antimicrobial susceptibility, although its clinical importance in odontogenic infections remains incompletely defined 8, 20, 21, 22. Increasing microbial burden and community complexity have been linked to polymicrobial synergy, ecological niche expansion, and enhanced selective pressure toward antimicrobial resistance 23, 24. Despite the inherently polymicrobial nature of odontogenic infections, this ecosystem-based perspective has seldom been applied in clinical research, and the independent contribution of immunosuppression to microbial complexity and resistance remains unclear.

Therefore, the aim of this study was to examine the relative associations of host immunosuppression and cultured microbial community complexity with polymicrobial infection, Enterobacterales detection, and antimicrobial resistance. This distinction is central for biologically grounded risk stratification and for optimizing empirical antibiotic selection. Given the retrospective observational design, the study was intended to identify associations rather than establish causal effects.

## Methods

**Fig. 1:**
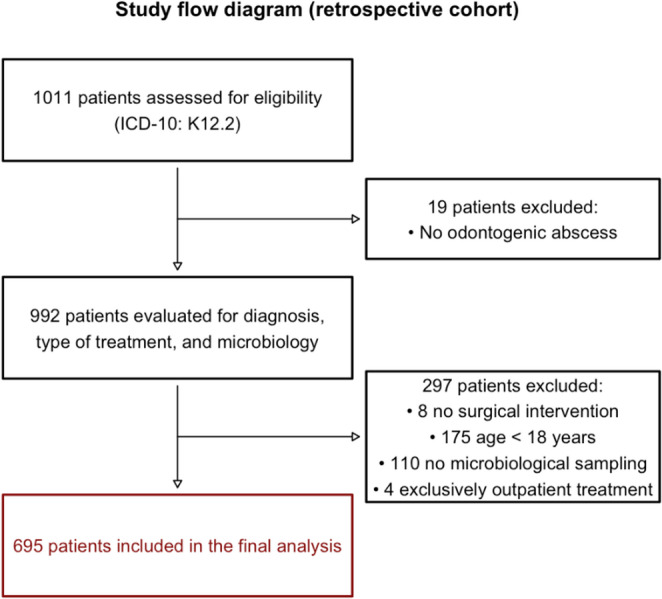
Study flow diagram of patient selection for the final cohort

This retrospective observational cohort study included all consecutive adult patients who underwent surgical treatment for odontogenic infections at a tertiary maxillofacial surgery center between 2017 and 2020. Eligible patients were required to be adults (≥ 18 years), to have a confirmed odontogenic origin of infection, to have received surgical drainage, and to have intraoperative microbiological cultures available; individuals without microbiological sampling were excluded, yielding a final cohort of 695 patients (Fig. 1). The study was approved by the local institutional ethics committee (approval no. EA2/002/26), and the need for written informed consent was waived owing to the retrospective design.

Clinical data were extracted from electronic medical records and included demographic variables, comorbidities, medication history, immune status, clinical presentation, laboratory parameters, radiologic findings, perioperative details, and postoperative course. Comorbidities were quantified using the Charlson Comorbidity Index (CCI), and preoperative physical status was assessed according to the American Society of Anesthesiologists (ASA) classification. Antibiotic exposure within 72 h before surgical incision was defined as prior systemic therapy. Infection severity was characterized by the number of affected anatomical compartments and the presence of systemic inflammatory signs.

Immunosuppressed status was assigned to patients receiving systemic glucocorticoids, disease-modifying antirheumatic drugs, biological immunosuppressive agents, active oncologic chemotherapy, as well as to solid organ transplant recipients and patients with documented HIV infection. All other individuals were classified as immunocompetent. This pragmatic definition combines biologically heterogeneous conditions.

Intraoperative specimens were obtained under sterile conditions and processed according to standardized institutional microbiology protocols, which included both aerobic and anaerobic cultures. Microbial identification was performed using routine laboratory methods including MALDI-TOF, and antimicrobial susceptibility testing was interpreted according to EUCAST standards. Identified microorganisms were categorized into predefined pathogen groups encompassing common odontogenic organisms such as streptococci, staphylococci, anaerobic bacteria including *Fusobacterium*,* Prevotella*, and *Actinomyces*, as well as *Enterobacterales* and *Candida species. Methicillin-resistant Staphylococcus aureus*,* vancomycin-resistant enterococci*, and multidrug-resistant gram-negative organisms were documented separately.

Three primary microbiological outcomes were defined prior to analysis: the presence of polymicrobial infection, detection of *Enterobacterales*, and detection of antimicrobial resistance. Secondary outcomes included the identification of *MRSA* and *Candida species*. Cultured microbial community complexity was quantified as the number of distinct pathogens detected per patient and was examined both as a binary variable distinguishing low (0–1 pathogen) from high (≥ 2 pathogens) complexity and as a three-level gradation (0–1, 2, or ≥ 3 pathogens) for dose–response analyses. Microbial community complexity was operationalized as the number of distinct pathogens detected by routine culture in each patient specimen. This variable was examined both dichotomously, comparing low complexity (0–1 pathogen) with higher complexity (≥ 2 pathogens), and ordinally (0–1, 2, or ≥ 3 pathogens) for dose–response analyses. This measure should be interpreted as a pragmatic culture-based richness metric and does not capture relative abundance, interspecies interactions, or functional ecological characteristics.

To explore compositional patterns of the microbial communities, unsupervised hierarchical clustering was performed using a pathogen presence–absence matrix, with Jaccard distance as the dissimilarity metric and complete linkage for clustering. Heatmaps were generated to visualize the resulting cluster structures.

Continuous variables were summarized as means with standard deviations or medians with interquartile ranges, depending on distributional properties, and categorical variables as absolute numbers and percentages. Group differences were evaluated using χ² tests or Fisher’s exact tests for categorical data and t-tests or Mann–Whitney U tests for continuous data. Statistical significance was defined as a two-sided p-value below 0.05.

To assess independent associations with the primary microbiological outcomes, separate multivariable logistic regression models were constructed for polymicrobial infection, *Enterobacterales* detection, and antimicrobial resistance. Each fully adjusted model included microbial complexity, immunosuppression, ASA classification, CCI, and prior antibiotic therapy. Results were expressed as adjusted odds ratios with 95% confidence intervals, and analyses were restricted to 359 complete cases. Sensitivity analyses were performed using reduced models that omitted ASA classification and CCI to avoid potential overadjustment; these were fitted in 695 patients. To evaluate biological gradient effects, additional logistic regression models incorporating the three-level microbial burden classification were used to examine dose–response relationships for *Enterobacterales* detection and antimicrobial resistance while adjusting for immunosuppression and prior antibiotic therapy. Fully adjusted multivariable models were performed as complete-case analyses. Patients with missing data in any covariate included in the respective model were excluded, resulting in 359 patients in the fully adjusted models. All statistical analyses were conducted using R (version 2025.05.0 + 496).

## Results

A total of 695 patients with surgically treated odontogenic infections and available microbiological cultures were included. Immunosuppressed patients were significantly older (64 [IQR 54–75] vs. 49 [IQR 36–65] years) and had a higher systemic disease burden, demonstrated by higher CCI scores (*p* < 0.001) and higher ASA classes (*p* < 0.001). In contrast, the number of involved anatomical compartments and the frequency of prior antibiotic therapy within 72 h before surgery did not differ between immunosuppressed and immunocompetent patients, indicating comparable initial local disease severity despite marked differences in host vulnerability.

Across the cohort, polymicrobial infection was detected in 62% of cases, and antimicrobial resistance in 40.6%. Neither polymicrobial infection (*p* = 0.64) nor overall antimicrobial resistance (*p* = 0.66) differed significantly between immunosuppressed and non-immunosuppressed patients. *MRSA* and *Candida spp*. were rare and showed no association with immune status. In contrast, *Enterobacterales* were detected significantly more frequently in immunosuppressed patients (13.9% vs. 3.9%, *p* < 0.001), indicating a selective gram-negative ecological shift without a corresponding increase in global resistance.

**Fig. 2:**
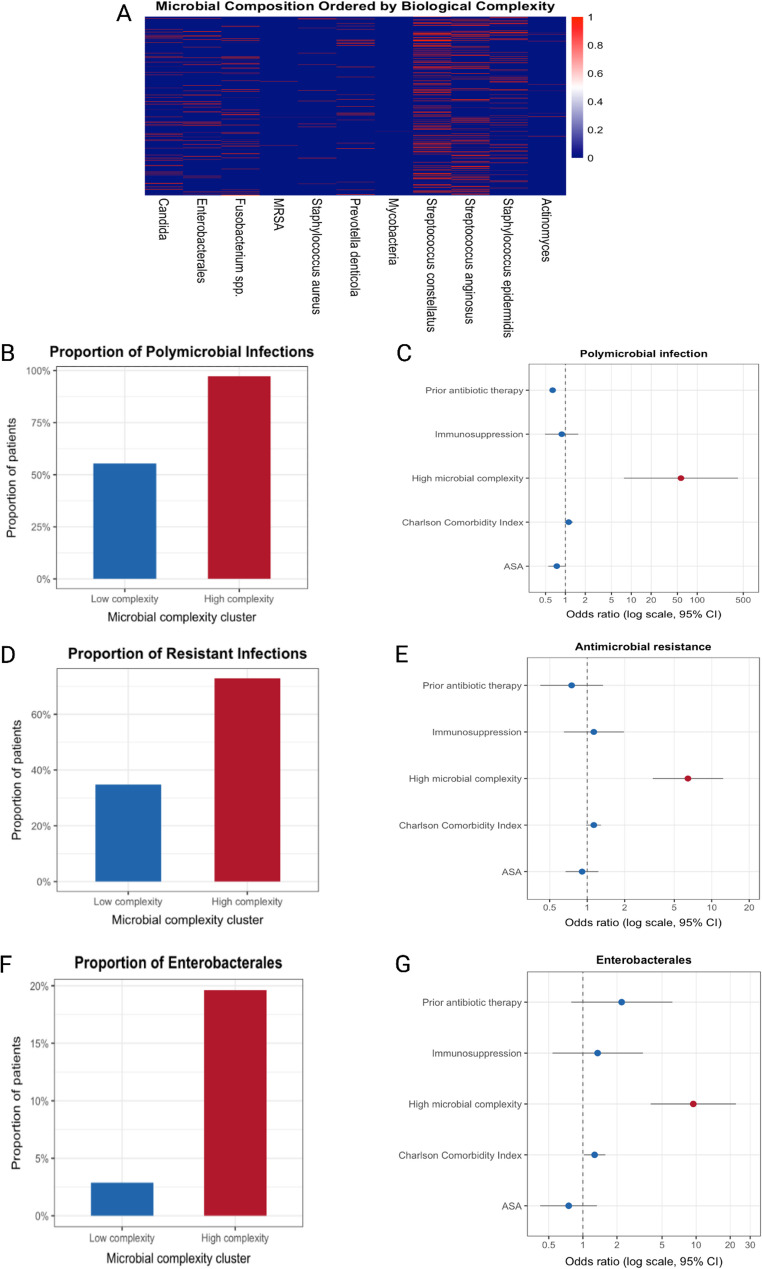
Cultured microbial community complexity and microbiological outcomes in odontogenic infections

To assess higher-order microbial organization, unsupervised hierarchical clustering of binary pathogen profiles identified two major microbial composition patterns **(**Fig. 2A**)**. Cluster membership showed no meaningful overlap with immune status, suggesting that immunosuppression does not determine global microbial ecosystem structure, and that clustering alone does not permit clinical risk stratification.

When microbial burden was defined biologically as the number of distinct pathogens detected per patient, a distinct high-complexity phenotype emerged (Fig. 2B–F). High microbial complexity (≥ 2 pathogens; *n* = 334) was associated with near-universal polymicrobial infection (97.2% vs. 55.3%, *p* < 0.001), markedly higher antimicrobial resistance (72.9% vs. 34.8%, *p* < 0.001), and substantial enrichment of *Enterobacterales* (19.6% vs. 2.9%, *p* < 0.001). The mean number of pathogens increased more than fivefold in high-complexity infections compared with low complexity (2.27 vs. 0.43, *p* < 0.001). Importantly, immunosuppressed and immunocompetent patients were distributed across low- and high-complexity groups, indicating that cultured microbial community complexity is fundamentally independent of host immune status.

In fully adjusted multivariable regression models (*n* = 359 complete cases), higher cultured microbial complexity showed the strongest adjusted association with all adverse microbiological outcomes **(**Fig. 2C, E and G). High microbial complexity significantly increased the odds of polymicrobial infection (OR 56.6, 95% CI 7.7–414.8; *p* < 0.001), Enterobacterales detection (OR 9.44, 95% CI 3.97–22.47; *p* < 0.001) and antimicrobial resistance (OR 6.44, 95% CI 3.36–12.34). No independent association was observed between the broad immunosuppression variable and polymicrobial infection (OR 0.88, 95% CI 0.49–1.56), *Enterobacterales* detection (OR 1.35, 95% CI 0.54–3.39) or antimicrobial resistance (OR 1.13, 95% CI 0.65–1.97) in the adjusted models. Among covariates, only CCI independently predicted *Enterobacterales* detection (OR 1.27 per point, 95% CI 1.02–1.58). Neither ASA classification nor prior antibiotic therapy predicted antimicrobial resistance (Supplementary Material 1). Age, sex, and smoking were examined in the initial multivariable models, but none showed a significant independent association that materially changed the main findings.

Sensitivity analyses excluding ASA and CCI (*n* = 695) confirmed the robustness of microbial complexity. High complexity remained strongly associated with polymicrobial infection (OR 28.12, 95% CI 8.81–89.78), *Enterobacterales* detection (OR 8.55, 95% CI 4.26–17.16), and antimicrobial resistance (OR 4.89, 95% CI 3.08–7.74). The broad immunosuppression variable and prior antibiotic therapy did not show statistically significant adjusted associations across the sensitivity models. (Supplementary Material 2).

Dose–response analyses demonstrated a clear biological gradient linking increasing pathogen burden to *Enterobacterales* detection and antimicrobial resistance. Compared with infections containing 0 − 1 pathogen, the presence of two pathogens significantly increased the likelihood of Enterobacterales detection (OR 7.86, 95% CI 3.70–16.72) and antimicrobial resistance (OR 4.42, 95% CI 2.66–7.33). Infections with ≥ 3 pathogens exhibited the strongest effects, with OR 11.04 (95% CI 3.81–32.02) for *Enterobacterales* detection and OR 7.14 (95% CI 2.64–19.36) for antimicrobial resistance. Immunosuppression did not retain independent significance in either model (Supplementary Material 3).

Taken together, these findings indicate that higher cultured microbial complexity was more strongly associated with polymicrobial infection, *Enterobacterales* detection, and antimicrobial resistance than the broad immunosuppression variable used in this study.

(A) Heatmap of pathogen distributions across the cohort (*n* = 695), ordered by increasing number of detected pathogens, illustrating a transition from sparse, low-diversity profiles (*n* = 361) to more compositionally complex culture profiles (*n* = 334). (B, D, F) Proportions of polymicrobial infection, antimicrobial resistance, and *Enterobacterales* detection stratified by cultured microbial complexity, showing enrichment of adverse microbiological phenotypes in higher-complexity infections. (C, E, G) Fully adjusted multivariable regression models (*n* = 359) showing cultured microbial complexity as the variable most strongly associated with all three primary outcomes, whereas no independent association was observed for the broad immunosuppression variable. Odds ratios are shown on a logarithmic scale with 95% confidence intervals.

## Discussion

In this large cohort of surgically treated odontogenic infections, we found that a higher number of cultured pathogens per patient was strongly associated with polymicrobial infection, *Enterobacterales* detection, and antimicrobial resistance. While immunosuppression exerted a selective effect on specific microbial niches, it did not independently determine overall resistance or polymicrobial burden. These findings suggest that community-level microbiological features may be relevant alongside host-related factors in odontogenic infection biology 8, 19 .

Immunosuppression is traditionally regarded as a major determinant of complicated infection courses and resistant pathogens, and empirical antibiotic escalation is frequently guided by immune status alone 25. Previous clinical studies have indeed reported higher complication rates and prolonged hospital stays in immunocompromised patients, yet microbiological evidence has remained inconsistent 1, 4, 5, 26, 27, 28. Some reports describe increased gram-negative or fungal detection, whereas others fail to demonstrate a clear association with antimicrobial resistance 2, 21. Our data add important granularity to this field by demonstrating that immunosuppression selectively modulates microbial composition, most notably by increasing the likelihood of *Enterobacterales* detection, without independently driving the global resistance phenotype. This suggests that impaired mucosal immunity, altered local host–microbe interactions, or increased healthcare exposure may facilitate the expansion of specific gram-negative niches. However, the absence of broader independent associations in our models should be interpreted cautiously given the heterogeneous composite definition of immunosuppression used in this study 22, 29 .

In contrast, microbial ecosystem complexity emerged as the most powerful and consistent predictor across all adverse microbiological endpoints. High-complexity infections were almost uniformly polymicrobial and exhibited a markedly increased prevalence of antimicrobial resistance together with pronounced *Enterobacterales* enrichment. These associations remained stable across fully adjusted models, sensitivity analyses, and graded dose–response testing, underscoring the robustness of microbial burden as a central biological risk determinant. This observation is well aligned with emerging concepts from microbial ecology and polymicrobial infection research, which increasingly emphasize that interspecies interactions, rather than single pathogens, govern virulence expression, metabolic cooperation, biofilm formation, and resistance selection 8, 19, 30, 31, 32, 33, 34 .

From a mechanistic perspective, odontogenic infections provide an ideal ecological framework for such synergistic processes. Anaerobic species such as *Fusobacterium*, *Prevotella*, and *Actinomyces* are known to shape local redox conditions, generate metabolic substrates for facultative pathogens, and facilitate biofilm maturation 34, 35, 36, 37. These processes can promote the expansion of facultative gram-negative bacteria and increase selective pressure toward antimicrobial resistance. The strong and graded association between increasing microbial burden and both *Enterobacterales* detection and antimicrobial resistance observed in our study provides quantitative clinical support for these experimentally derived concepts 30, 31, 38, 39, 40, 41 .

A particular strength of the present analysis is the demonstration of a clear biological dose–response relationship. The stepwise increase in resistance and gram-negative enrichment across increasing levels of pathogen burden supports the internal consistency and biological plausibility of the observed associations. However, given the retrospective observational design, these gradients should not be interpreted as establishing causality 42. Importantly, this gradient persisted after adjustment for immunosuppression and prior antibiotic exposure, suggesting that the observed associations were not fully explained by host vulnerability or recent treatment exposure.

These findings have potential clinical relevance, but they do not yet translate directly into empirical treatment decisions. Because microbial complexity as defined here was derived from culture results, it is generally not available at the time initial antibiotic therapy is chosen. The more meaningful implication is therefore that microbiological risk in odontogenic infections may not be adequately captured by host status alone^5, 27, 43, 44, 45, 46^. Rather than supporting immediate changes in empirical therapy, our data support further work on faster microbiological or molecular approaches that could make community-level risk information clinically usable. These findings also do not imply that culture-derived complexity should guide the indication for surgery, which remains based on established clinical criteria such as severity, anatomical spread, and airway risk. However, they may still be relevant for future microbiological risk stratification in surgically treated patients.

Several limitations should be acknowledged. The retrospective single-center design limits causal inference and generalizability, particularly because the cohort was restricted to surgically treated cases at a tertiary center. In addition, the key exposure variable was derived from routine culture and should therefore be interpreted as a pragmatic culture-based richness measure rather than a comprehensive assessment of ecological complexity. Because culture may underestimate true diversity and is influenced by prior antibiotic exposure, sampling conditions, and organism-specific growth requirements, the measured complexity may partly reflect detectability rather than the underlying microbial structure. Residual confounding cannot be excluded. Conceptual overlap between pathogen count–based complexity and polymicrobial infection may partly explain the large effect size for that endpoint, and the broad, biologically heterogeneous definition of immunosuppression may have reduced sensitivity to detect subgroup-specific associations. Nevertheless, the large cohort and internal consistency across analyses support the robustness of the observed associations. These findings also do not imply that culture-derived complexity should guide the indication for surgery, which remains based on established clinical criteria such as severity, anatomical spread, and airway risk. However, they may still be relevant for future microbiological risk stratification in surgically treated patients.

In summary, this study suggests that higher cultured microbial complexity is strongly associated with adverse microbiological phenotypes in surgically treated odontogenic infections. Immunosuppression selectively modulates specific microbial niches but does not independently define the global resistance phenotype. These results support a broader view of microbiological risk in odontogenic infections that extends beyond host status alone and provide a rationale for future risk stratification approaches incorporating community-level microbial features. 

## Supplementary Information

Below is the link to the electronic supplementary material.

Supplementary Material 1 (DOCX 21.7 KB)

## Data Availability

All data supporting the findings of this study are available within the paper and its Supplementary Information.
