# Supplementary material for: Cultured microbial community complexity is associated with antimicrobial resistance and Enterobacterales enrichment in adult odontogenic infections
Source: Oral Maxillofac Surg. 2026 Apr 24;30(1):76. doi: 10.1007/s10006-026-01563-3 (PMC13109227; doi:10.1007/s10006-026-01563-3)
Supplement: Supplementary file 1 — Supplementary Material 1 (DOCX 21.7 KB) [file 10006_2026_1563_MOESM1_ESM.docx]

**Microbial ecosystem complexity is associated with antimicrobial resistance and Enterobacterales enrichment in odontogenic infections**

A. Schmitz^1*^, N. L. Kern^1^, H. Ward^1^, M. Sauerbrey^1^, F. Mrosk^1^, O. Wagendorf^1^, S. Nahles^1^, C. Rendenbach^1^, N. Neckel^1^, M. Heiland^1^, S. Koerdt^1^

^1^ Charité – Universitätsmedizin Berlin, corporate member of Freie Universität Berlin and Humboldt-Universität zu Berlin, Department of Oral and Maxillofacial Surgery, Augustenburger Platz 1, 13353 Berlin, Germany

* **Corresponding author**: Alina Marie Schmitz

Department of Oral and Maxillofacial Surgery,

Charité *–* Universitätsmedizin Berlin, Augustenburger Platz 1, 13353 Berlin, Germany

Email: [alina-marie.schmitz@charite.de](mailto:alina-marie.schmitz@charite.de)

Tel: +49 30 450 655 014

Fax: +49 30 450 555901

Supplementary Material

# Supplementary Material 1: Multivariable Regression Analyses

## Fully Adjusted Model – Polymicrobial Infection

| Variable | OR | 95% CI (Lower) | 95% CI (Upper) | P-value |
| --- | --- | --- | --- | --- |
| Microbial complexity (High) | 56.59 | 7.72 | 414.78 | 7.16e-05 |
| Immunosuppression | 0.88 | 0.49 | 1.56 | 0.654 |
| ASA classification | 0.74 | 0.55 | 1.01 | 0.062 |
| Charlson Comorbidity Index | 1.12 | 0.97 | 1.3 | 0.116 |
| Prior antibiotic therapy | 0.64 | 0.37 | 1.12 | 0.119 |

## Fully Adjusted Model – Enterobacterales Detection

| Variable | OR | 95% CI (Lower) | 95% CI (Upper) | P-value |
| --- | --- | --- | --- | --- |
| Microbial complexity (High) | 9.44 | 3.97 | 22.47 | <0.001 |
| Immunosuppression | 1.35 | 0.54 | 3.39 | 0.521 |
| ASA classification | 0.75 | 0.42 | 1.33 | 0.323 |
| Charlson Comorbidity Index | 1.27 | 1.02 | 1.58 | 0.032 |
| Prior antibiotic therapy | 2.2 | 0.79 | 6.17 | 0.133 |

## Fully Adjusted Model – Antimicrobial Resistance

| Variable | OR | 95% CI (Lower) | 95% CI (Upper) | P-value |
| --- | --- | --- | --- | --- |
| Microbial complexity (High) | 6.44 | 3.36 | 12.34 | <0.001 |
| Immunosuppression | 1.13 | 0.65 | 1.97 | 0.656 |
| ASA classification | 0.91 | 0.67 | 1.23 | 0.546 |
| Charlson Comorbidity Index | 1.13 | 0.98 | 1.29 | 0.086 |
| Prior antibiotic therapy | 0.75 | 0.42 | 1.34 | 0.328 |

## Supplementary Material 2: Sensitivity Models

## Sensitivity Model – Polymicrobial Infection

| Variable | OR | 95% CI (Lower) | 95% CI (Upper) | P-value |
| --- | --- | --- | --- | --- |
| Microbial complexity (High) | 28.120532402617897 | 8.807397558508862 | 89.7841090235245 | <0.001 |
| Immunosuppression | 0.7499115595537207 | 0.4475943166549407 | 1.2564220014121261 | 0.274 |
| Prior antibiotic therapy | 0.8032413899982859 | 0.5405241294870926 | 1.1936501913774145 | 0.278 |

## Sensitivity Model – Enterobacterales Detection

| Variable | OR | 95% CI (Lower) | 95% CI (Upper) | P-value |
| --- | --- | --- | --- | --- |
| Microbial complexity (High) | 8.54887760446619 | 4.258938709422971 | 17.15998122594382 | <0.001 |
| Immunosuppression | 2.2805120475265084 | 0.9929926666594843 | 5.237435656407499 | 0.052 |
| Prior antibiotic therapy | 1.773934569190453 | 0.7804816889250993 | 4.031925284631389 | 0.171 |

## Sensitivity Model – Antimicrobial Resistance

| Variable | OR | 95% CI (Lower) | 95% CI (Upper) | P-value |
| --- | --- | --- | --- | --- |
| Microbial complexity (High) | 4.887104493543578 | 3.0838425482372993 | 7.744815099093056 | <0.001 |
| Immunosuppression | 1.1968103784096704 | 0.7307730007114854 | 1.9600547372091581 | 0.475 |
| Prior antibiotic therapy | 0.7623267571903688 | 0.5079489137219375 | 1.1440955360454093 | 0.190 |

## Supplementary Material 3: Dose-Response Models

## Dose–Response Model – Antimicrobial Resistance

| Variable | OR | 95% CI (Lower) | 95% CI (Upper) | P-value |
| --- | --- | --- | --- | --- |
| 2 pathogens vs. 0–1 | 4.42 | 2.66 | 7.33 | <0.001 |
| ≥3 pathogens vs. 0–1 | 7.14 | 2.64 | 19.36 | 0.000111 |
| Immunosuppression | 1.19 | 0.73 | 1.95 | 0.489 |
| Prior antibiotic therapy | 0.77 | 0.51 | 1.15 | 0.197 |

## Dose–Response Model – Enterobacterales Detection

| Variable | OR | 95% CI (Lower) | 95% CI (Upper) | P-value |
| --- | --- | --- | --- | --- |
| 2 pathogens vs. 0–1 | 7.86 | 3.7 | 16.72 | <0.001 |
| ≥3 pathogens vs. 0–1 | 11.04 | 3.81 | 32.02 | <0.001 |
| Immunosuppression | 2.26 | 0.98 | 5.19 | 0.056 |
| Prior antibiotic therapy | 1.8 | 0.79 | 4.09 | 0.162 |
